# Deficiency of ACE2 in Bone-Marrow-Derived Cells Increases Expression of TNF-**α** in Adipose Stromal Cells and Augments Glucose Intolerance in Obese C57BL/6 Mice

**DOI:** 10.1155/2012/762094

**Published:** 2012-02-06

**Authors:** Sean E. Thatcher, Manisha Gupte, Nicholas Hatch, Lisa A. Cassis

**Affiliations:** Graduate Center for Nutritional Sciences, University of Kentucky, Lexington, KY 40536-0200, USA

## Abstract

Deficiency of ACE2 in macrophages has been suggested to promote the development of an inflammatory M1 macrophage phenotype. We evaluated effects of ACE2 deficiency in bone-marrow-derived stem cells on adipose inflammation and glucose tolerance in C57BL/6 mice fed a high fat (HF) diet. ACE2 activity was increased in the stromal vascular fraction (SVF) isolated from visceral, but not subcutaneous adipose tissue of HF-fed mice. Deficiency of ACE2 in bone marrow cells significantly increased mRNA abundance of F4/80 and TNF-**α** in the SVF isolated from visceral adipose tissue of HF-fed chimeric mice, supporting increased presence of inflammatory macrophages in adipose tissue. Moreover, deficiency of ACE2 in bone marrow cells modestly augmented glucose intolerance in HF-fed chimeric mice and increased blood levels of glycosylated hemoglobin. In summary, ACE2 deficiency in bone marrow cells promotes inflammation in adipose tissue and augments obesity-induced glucose intolerance.

## 1. Introduction

Angiotensin-converting enzyme-2 (ACE2) is a monocarboxypeptidase which is responsible for converting angiotensin II (AngII) to angiotensin 1–7 (Ang-(1–7)). Previous studies demonstrated expression of a complete renin-angiotensin system (RAS) in bone marrow cells, including renin, angiotensin converting enzyme (ACE), ACE2, AngII, and angiotensin receptors (AT1 and AT2) [1, 2]. Recent studies in our laboratory demonstrated ACE2 enzymatic activity in macrophages and localization of ACE2 immunoreactivity to macrophage-containing atherosclerotic lesions [2]. Moreover, deficiency of ACE2 in bone-marrow-derived stem cells promoted the development of diet-induced atherosclerosis in low-density-lipoprotein-receptor (LDLR-)deficient mice [2]. Peritoneal macrophages from ACE2-deficient LDLR−/− mice exhibited increased release of AngII, IL-6, and plasminogen activator inhibitor-1 (PAI-1), and conditioned media from ACE2-deficient macrophages promoted monocyte adhesion to endothelial cells [2]. These results suggest that elevated levels of AngII in ACE2-deficient leukocytes may promote adhesion of monocytes to vascular endothelial cells. Using bone-marrow-derived macrophages from mice with combined deficiency of apolipoprotein E and ACE2, Thomas et al. demonstrated enhanced lipopolysaccharide-(LPS-) induced mRNA abundance of tumor necrosis factor-alpha (TNF*α*), monocyte chemoattractant protein-1 (MCP-1), interleukin-6 (IL-6), and matrix metalloproteinase-9 (MMP-9) [3]. In addition, the mas receptor was localized to peritoneal macrophages and Ang-(1–7) decreased LPS-induced inflammatory responses [4]. Collectively, these results suggest that macrophage ACE2 influences levels of AngII/Ang-(1–7), potentially contributing to macrophage-mediated inflammation.

Obesity is known to increase macrophage infiltration into adipose tissue, and adipose tissue macrophages (ATMs) are mainly derived from the bone marrow [5, 6]. Infiltration of macrophages with obesity promotes inflammation in adipose tissue and has been linked to the development of insulin resistance and type 2 diabetes [7]. Adipocytes secrete a number of different cytokines that can influence the polarization state of macrophages in adipose tissue. Macrophages that are recruited to adipose tissue display an activated state, termed M1 polarization [8–10]. Activated M1 macrophages have increased expression of IL-6, inducible nitric oxide (iNOS), and C-C chemokine receptor 2 (CCR2) [8]. Alternatively activated macrophages (M2 polarization) counterbalance the proinflammatory status in adipose tissue [9]. Since peritoneal macrophages from ACE2-deficient mice displayed increased expression and/or release of several M1 macrophage-related cytokines, macrophages from ACE2-deficient mice have been suggested to exhibit M1 polarization [2].

Previous studies demonstrated that diet-induced obesity is associated with an activated systemic and adipose renin-angiotensin system (RAS) [11, 12]. Increased plasma concentrations of AngII in male C57BL/6 mice with diet-induced obesity were associated with dysregulated ACE2 in adipose tissue and the development of obesity hypertension [12]. These results suggest that obesity is associated with changes in the adipose RAS, including ACE2. Since obesity is associated with increased macrophage infiltration into adipose tissue, the specific cell type(s) experiencing previously observed alterations in ACE2 function in adipose tissue of obese mice is unclear [12]. Moreover, the role of macrophage-derived ACE2 in obesity-induced inflammation of adipose tissue has not been defined. Bone marrow transplantation in irradiated mice has been extensively employed to define the role of leukocytes in various disease pathologies. The purpose of this study was to define the effect of leukocyte deficiency of ACE2, using bone marrow transplantation from ACE2-deficient mice, on the development of obesity, adipose inflammation, and glucose intolerance in high-fat-(HF-) fed C57BL/6 mice.

## 2. Methods

### 2.1. Mice and Bone Marrow Transplantation

All experiments involving mice conformed to the National Institutes of Health Guide for the Care and Use of Laboratory Animals and were approved by the University of Kentucky Institutional Animal Care and Use Committee. Male, 8-week-old C57BL/6 mice were purchased from Jackson Labs (Bar Harbor, MA) and housed in a temperature-controlled room with a 12 : 12-h light-dark cycle. *Ace2^+/y^* or *Ace2^−/y^* C57BL/6 mice were 10-times backcrossed onto a C57BL/6 background [13]. Initial studies examined ACE2 activity in the stromal vascular fraction (SVF) isolated from adipose tissues of male C57BL/6 mice (2 months of age; *N* = 10  /group) fed a low fat (LF; 10% kcal as fat, D12450, Research Diets Inc, New Brunswick, NJ) or high fat (HF, 60% kcal as fat, D12492, Research Diets Inc, New Brunswick, NJ) diet for 16 weeks. For bone marrow transplantation, C57BL/6 male mice (2 months of age) were pretreated with antibiotic water (sulfatrim, 4 *μ*g/mL) for 1 week prior to irradiation [2, 14]. Bone marrow was extracted from the tibias and femurs of *Ace2^+/y^* or *Ace2^−/y^* male mice (2 months of age) and injected into gamma-irradiated recipient C57BL/6 males (*N* = 15 mice/donor genotype) at a dose of 10^7^ cells per mouse. Recipient mice were given antibiotic water for 8 weeks to allow for efficient repopulation [14]. Mice in each donor genotype were fed the HF diet for 4 months. Body weight was recorded weekly. To define fat/lean mass, Dual Energy X-ray Absorptiometry (DEXA) was performed on anesthetized mice prior to initiation of the HF diet and at study endpoint. At study endpoint, mice were anesthetized (ketamine/xylazine 100/10 mg/kg, ip) for exsanguination to obtain blood for white cell counts (WBCs, K indicates 1000 per microliter), hemoglobin concentration (grams per deciliter), and bone marrow (femur) was harvested to confirm effective bone marrow repopulation (data not shown).

### 2.2. Measurement of Plasma and Serum Parameters

Fasting (6 hr) blood glucose concentrations (mg/dL) were measured with a glucometer (FreeStyle Strips, Abbott Labs, Alameda, CA) at 1, 2, and 3 months of HF-feeding. During month 4 of HF feeding, a glucose tolerance test (GTT) was performed on fasted (6 hr) mice. Blood glucose concentrations were quantified at 0, 15, 30, 60, 90, 125, 160, and 220 minutes after glucose administration (2 mg/g glucose, ip). Percent glycosylated hemoglobin (%GHb) was quantified in whole blood according to the manufacturer's instructions (Glycohemoglobin Reagent Set-Unitized, cat no. G7540-100, Pointe Scientific, Inc., Canton MI). Plasma concentrations of insulin were quantified in nonfasted mice by ELISA according to the manufacturer's instructions (Millipore Inc., Billerica, MA). Serum concentrations of cholesterol, triglyceride, and free fatty acids were quantified using colorimetric kits from Wako Pure Chemical Industries (Osaka, Japan). Serum ACE activity (Table 1) was quantified using 5 mM N-Hippuryl-His-Leu as a substrate in 0.4 M sodium borate, pH 8.3 (30 min incubation at 37°C), with 2% o-phthaldialdehyde added to measure the fluorescence of the reaction for 10 minutes at room temperature. Reactions were preincubated with and without captopril (1 *μ*M) for 30 minutes at 37°C to assess specificity. Absorbance was measured at an excitation of 365 nm and an emission of 495 nm. Specific activity was normalized to total volume of serum added to the reaction (2 *μ*Ls). Plasma renin concentrations were quantified by incubating mouse plasma (8 *μ*Ls) with an excess of partially purified rat angiotensinogen (from nephrectomized rats) in the presence of ACE inhibition (EDTA, captopril, 1 *μ*M), followed by quantification of angiotensin I concentrations by radioimmunoassay (DiaSorin CA-1553, Stillwater, MN) [15]. To quantify plasma concentrations of AngII, plasma was first processed over mini-C18 columns to concentrate peptides, followed by quantification of AngII by radioimmunoassay using an anti-rabbit AngII antibody (1 : 40,000 dilution; Bachem, Torrance, CA) that exhibits cross reactivity to AngIII (100%), AngIV (75%), but minimal reactivity to other angiotensins [14, 16].

### 2.3. Isolation of Stromal Vascular Fraction (SVF) from Adipose Tissue and Quantification of ACE2 Enzymatic Activity

 Adipose tissue (epididymal fat, EF; retroperitoneal fat, RPF; subcutaneous fat, SubQ) was minced and digested with Type I collagenase (1 mg/mL; 60 min at 37°C) in buffer containing fatty acid-free bovine serum albumin (1%) [17]. Digested material was filtered (100 *μ*m nylon mesh) and centrifuged (500 g) for 10 minutes to pellet SVF (frozen at −70°C unless used for ACE2 activity). ACE2 enzymatic activity was quantified in SVF by examining the conversion of [^125^I]-AngII to [^125^I]-Ang-(1–7) [2, 12]. Briefly, SVF was homogenized in Tris buffer (100 mM) containing NaCl (0.3 M), ZnCl_2_ (10 *μ*M), and Z-proprolinal (10 *μ*M). Following centrifugation (30,000 g for 20 minutes, 4°C), pellets were reconstituted in the above buffer containing 0.5% Triton-X and incubated overnight at 4°C. Samples were again centrifuged (5,000 g for 10 minutes, 4°C) and the supernatant containing solubilized membrane was used for measurement of protein (BCA assay, ThermoFischer) and ACE2 enzymatic activity. SVF protein (0.05 mg/mL) was added to tubes with Tris buffer (total volume was 250 *μ*Ls) containing the following inhibitors: thiorphan (0.1 mM), phosphoramidon (0.1 mM), bestatin (100 *μ*M), pepstatin A (100 *μ*M), and captopril (10 *μ*M) (pH = 7.0). [^125^I]-AngII (2 × 10^6^ cpms) was incubated with samples for 30 minutes at 37°C and the reactions were stopped by adding 50 *μ*Ls of 1% phosphoric acid. Samples were centrifuged, filtered, and injected onto a Beckman reverse-phase HPLC to resolve angiotensins [12]. Retention times for [^125^I]-Ang-(1–7) (6.6 minutes) and [^125^I]-AngII (13.6 minutes) were used to define HPLC fractions containing angiotensins, and radioactivity was quantified by gamma counting. ACE2 activity is expressed as femtomoles per milligram protein per minute, based on the specific activity of [^125^I]-AngII [12].

### 2.4. Quantification of mRNA Abundance by RT-PCR

SVF pellets were placed in RNA lysis buffer (Promega, Madison, WI), and RNA was extracted using an SV Total RNA isolation kit (Promega, Madison, WI). RNA absorbance was measured at 260/280 nm and reverse transcription reactions were conducted on 0.5 *μ*g of total RNA. Reverse transcription reactions were performed using a RETROscript kit (Ambion Inc, Austin, TX) through use of random decamers and heat denaturation of the RNA. Subsequent PCR analysis was performed using SYBR Green PCR core reagents (Applied Biosystems, Foster City, CA), and real-time conditions were as follows: 2.5 minutes at 95°C, 40 cycles of 1 minute at 94°C, 1 minute at reannealing temperature, 1 minute at 72°C, and a final elongation step at 72°C for 10 minutes. Cyclophilin A was used to control for loading material and mRNA abundance was quantified using the 2^-ΔΔCt^ method. Primers for specific genes are listed in Table 2. 

### 2.5. Statistical Analysis

Data are expressed as mean ± SEM. All data were analyzed using Sigma Stat. For one factor analysis (diet or genotype), a *t*-test was used to analyze end-point measures. For GTTs, a repeated measures two-way ANOVA was performed with a Holm-Sidak test for multiple comparisons. Significance was accepted at *P* > 0.05.

## 3. Results

### 3.1. ACE2 Activity is Increased in SVF from Adipose Tissue of HF-fed Mice

**Figure 1:**
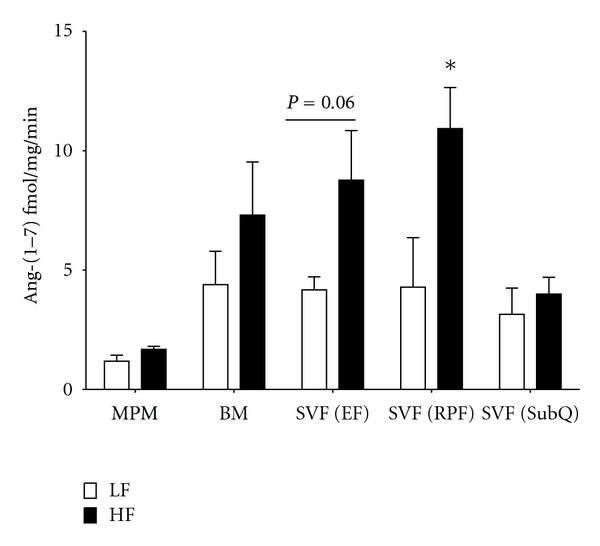
ACE2 activity is increased in the stromal vascular fraction (SVF) isolated from visceral adipose tissue of HF-fed C57BL/6 mice. ACE2 activity was quantified in mouse peritoneal macrophages (MPMs), bone marrow (BM), and SVF isolated from epididymal (EF), retroperitoneal (RPF) or subcutaneous (SubQ) adipose tissue from LF or HF-fed mice. Data are mean ± SEM from *N* = 3-4 mice/diet group. *: *P* < 0.05 compared to LF.

We quantified the effect of HF feeding on ACE2 activity in peritoneal macrophages (MPM), bone marrow (BM), and the SVF isolated from different adipose depots of LF and HF-fed mice. In MPMs and BM, ACE2 activity was not influenced by HF feeding. In contrast, the SVF isolated from visceral (RPF) adipose tissue, but not gonadal (EF) or subcutaneous (SubQ) adipose tissue of HF-fed mice, exhibited significantly increased ACE2 activity compared to LF-fed controls (Figure 1; *P* < 0.05 for RPF; *P* = 0.06 for EF; *P* > 0.05 for SubQ). 

### 3.2. Bone Marrow Deficiency of ACE2 Promotes Increases in Macrophage and Inflammatory Markers and Glucose Intolerance in HF-fed Mice

Deficiency of ACE2 in bone-marrow-derived cells had no significant effect on body weight or fat mass in HF-fed mice (Table 1). In addition, ACE2 deficiency in bone marrow cells had no significant effect on several plasma parameters measured in HF-fed mice (Table 1). Plasma renin (*P* = 0.06) or AngII concentrations (*P* = 0.148) were not statistically different between groups indicating that the systemic RAS was not influenced by the bone marrow repopulation. Bone marrow transplantation had no effect on WBC cell counts (WBC: *Ace2^+/y^*, 3.9 ± 1; *Ace2^−/y^*,  3.7 ± 0.2 K/*μ*L). However, irradiated mice receiving donor marrow from each genotype exhibited a significant reduction in blood hemoglobin (normal range 11–15 g/dL), but there were no differences between genotypes (*Ace2^+/y^*, 7.6 ± 0.6; *Ace2^−/y^*,  8.7 ± 0.6 g/dL, *P* = 0.22).

**Figure 2:**
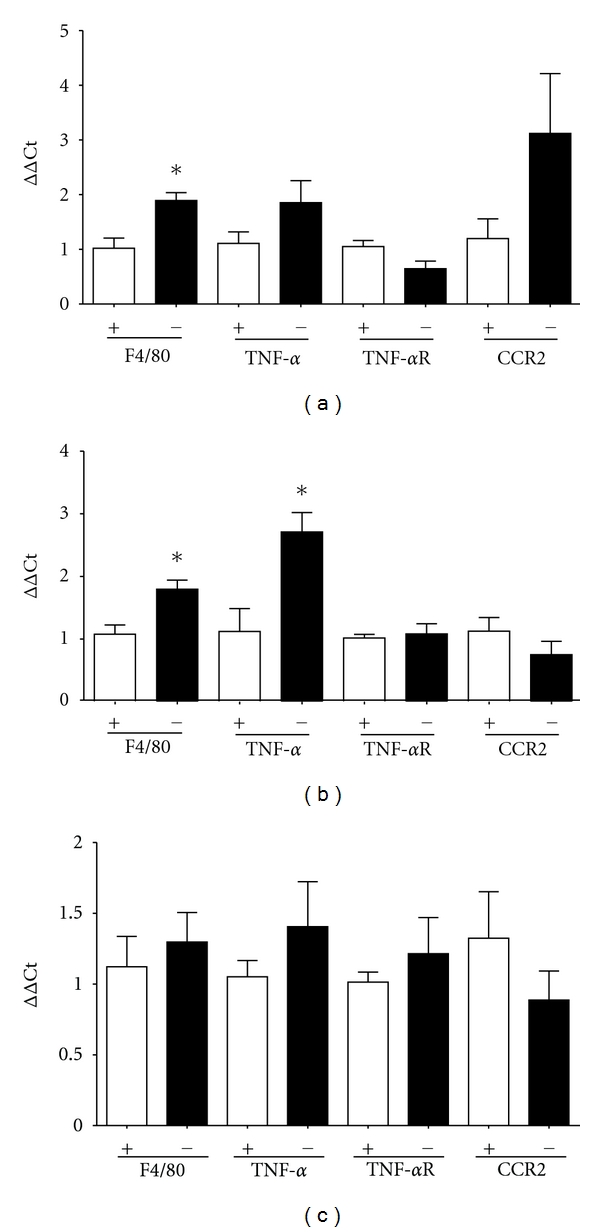
Bone marrow deficiency of ACE2 increases F4/80 and TNF-*α* mRNA abundance in the stromal vascular fraction (SVF) isolated from visceral adipose tissue of HF-fed C57BL/6 mice. Quantification of mRNA abundance in the SVF isolated from epididymal adipose (EF, a), retroperitoneal adipose (RPF, b), or subcutaneous fat (SubQ, c) of chimeric mice transplanted with *Ace2^+/y^* (depicted on the *x*-axis as +) or *^−/y^* (depicted on the *x*-axis as−) bone marrow. Data are mean ±  SEM from *N* = 3–11 mice/donor genotype. *: *P* < 0.05 compared to +.

To define effects of ACE2 deficiency in leukocytes on expression of M1 polarization markers in adipose tissue, we quantified mRNA abundance in the SVF isolated from EF (Figure 2(a)), RPF (Figure 2(b)), and SubQ (Figure 2(c)) of HF-fed mice transplanted with *Ace2^+/y^* or *^−/y^* bone marrow. Expression of the macrophage marker, F4/80, was significantly increased in the SVF isolated from EF and RPF from HF-fed mice transplanted with bone marrow from *Ace2^−/y^* compared to *^+/y^* mice (Figures 2(a) and 2(b); *P* < 0.05). In contrast, deficiency of ACE2 in leukocytes had no significant effect on F4/80 mRNA abundance in the SVF isolated from SubQ tissue of HF-fed chimeric mice (Figure 2(c)). In the SVF isolated from RPF of chimeric *Ace2^−/y^*mice, mRNA abundance of TNF-*α* was significantly increased compared to controls. However, other M1 markers (CCR2, EF, *P* = 0.282, TNF*α* receptor, Figures 2(a)–2(c); IL-6, IL-1*β*, CCL2, PAI-1, Mgl-1(data not shown)) did not exhibit significant differences in the SVF isolated from chimeric *Ace2^−/y^* compared to *^+/y^* mice. We also quantified M2 markers (IL-4, Ym-1, and Mrc-1) with no significant differences between genotypes (data not shown).

**Figure 3:**
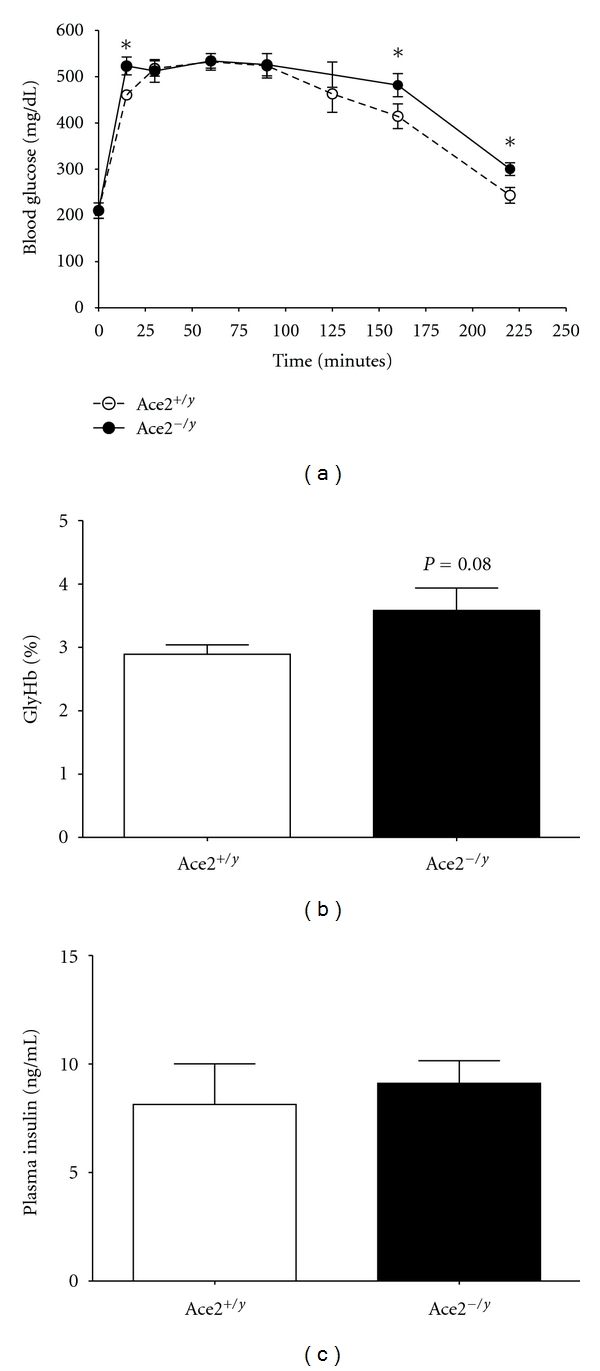
Bone marrow deficiency of ACE2 augments glucose intolerance at specific time points following glucose injection in HF-fed C57BL/6 mice. (a) Glucose tolerance tests (GTT) in HF-fed chimeric mice transplanted with bone marrow from *Ace2^+/y^* or *^−/y^* mice. (b) Percent glycosylated hemoglobin levels and plasma insulin concentrations (c) in *Ace2^+/y^* and *^−/y^* chimeric mice. Data are mean ± SEM from *N* = 8–15  mice/donor genotype. *: *P* < 0.05 compared to *Ace2^+/y^*.

Chimeric mice transplanted with *Ace2^−/y^* bone marrow exhibited significantly increased blood glucose concentrations at 15, 160, and 220 minutes after a glucose challenge compared to mice transplanted with *Ace2^+/y^* bone marrow (Figure 3(a); *P* < 0.05). In addition, blood levels of glycosylated hemoglobin were modestly, but not significantly, increased in plasma from *Ace2^−/y^* chimeric mice (*Ace2^+/y^*, 2.9 ± 0.1; *Ace2^−/y^*, 3.6 ± 0.4%, Figure 3(b); *P* = 0.08). In contrast, plasma concentrations of insulin were not significantly different between groups (*Ace2^+/y^*, 8 ± 2*; Ace2^−/y^*, 9 ± 1 ng/mL, *P* = 0.66).

## 4. Discussion

Results from this study demonstrate that deficiency of ACE2 in leukocytes promotes inflammation in adipose tissue and results in a modest impairment of glucose tolerance in obese mice. Initial studies identified an increase in ACE2 activity in heterogeneous stromal cells isolated from visceral adipose tissue, but not in more purified populations of peritoneal or bone marrow macrophages from HF compared to LF-fed mice. Notably, deficiency of ACE2 in bone-marrow-derived cells increased F4/80 expression in the SVF of RPF tissue, suggesting increased infiltration of macrophages into adipose tissue of chimeric mice lacking ACE2 in leukocytes. Moreover, expression of TNF-*α* was increased in the SVF of RPF tissue from chimeric mice lacking ACE2 in bone marrow, supporting increased adipose inflammation from deficiency of ACE2. Finally, obesity-induced impairment of glucose homeostasis was modestly augmented in chimeric ACE2-deficient mice. These results suggest that deficiency of ACE2 in leukocytes promotes adipose tissue inflammation and augments the development of obesity-induced diabetes.

Initial studies examined effects of diet-induced obesity on ACE2 activity in macrophages isolated from the peritoneal cavity, bone marrow, or the stromal vascular fraction of adipose tissue from different regions. Of these different cell isolations, the SVF included several other cell types in addition to macrophages. Notably, ACE2 activity was increased by HF-feeding in the SVF isolated from visceral, but not subcutaneous adipose tissue. Deposition of excess adipose tissue in the visceral cavity has been linked to several obesity-associated diseases, including hypertension and diabetes [18, 19]. Mechanisms for increased risk from visceral adipose accumulation are not clear, but may relate to increased metabolic activity of visceral adipose tissue [20]. Region-specific effects of obesity to increase visceral, but not subcutaneous ACE2 activity, are consistent with increased metabolic activity and a more pronounced impact of visceral adipose tissue on obesity-associated diabetes. However, since increased ACE2 activity would predictably lower local levels of AngII, these results suggest that activated ACE2 may serve as a compensatory protective mechanism. Alternatively, since F4/80 mRNA abundance was increased in visceral adipose tissue from HF-fed mice, increased ACE2 activity in the SVF may have resulted from increased macrophage infiltration to visceral adipose tissue of HF-fed mice. This finding is consistent with results from studies demonstrating that obesity is associated with more pronounced infiltration of macrophages in visceral compared to subcutaneous adipose tissue [21]. Moreover, this conclusion is supported by data demonstrating that ACE2 activity in more purified populations of macrophages, such as peritoneal macrophages or the bone marrow, was not increased by HF feeding. Thus, increased ACE2 activity in the stromal vascular fraction of HF-fed mice most likely arose from increased macrophage infiltration to adipose tissue.

Given findings of increased ACE2 activity in the SVF from visceral adipose tissue of HF-fed mice, we quantified effects of ACE2 deficiency in infiltrating leukocytes on adipose inflammation and glucose homeostasis using bone marrow transplantation. Bone marrow transplantation has been previously employed to define the source of macrophages infiltrating into adipose tissue with obesity [5] and by our laboratory to determine the role of leukocyte components of the RAS on developing atherosclerotic lesions [2, 14]. Our results do not support a role for leukocyte ACE2 in the development of obesity in HF-fed mice. In addition, consistent with previous studies, [2] systemic concentrations of cholesterol and/or fatty acids were not influenced by ACE2 deficiency in leukocytes. Moreover, deficiency of ACE2 in bone-marrow-derived stem cells had no effect on systemic concentrations of renin or AngII, similar to previous studies examining effects of renin deficiency in bone-marrow-derived cells on atherosclerosis in western diet-fed LDLR−/− mice [22].

A novel finding in this study was that ACE2 deficiency in bone-marrow-derived stem cells increased mRNA abundance of a macrophage marker (F4/80) and TNF-*α* in the SVF from visceral adipose tissue. As described above, increased mRNA abundance of F4/80 in the SVF from ACE2 chimeric mice supports increased infiltration of macrophages into adipose tissue. Increased infiltration of macrophages into adipose tissue was associated with elevated expression of TNF-*α*, a marker of M1 polarized macrophages. TNF-*α* has a well-defined role in the development of insulin resistance in both mice and humans [23–26]. Moreover, previous studies demonstrated that AngII increases secretion of TNF-*α* from RAW 264.7 macrophages [27] and that blockade of AT1 receptors in bone marrow stromal cells decreased TNF-*α* mRNA abundance [28]. Thus, it is conceivable that increased levels of AngII released from macrophages of ACE2-deficient mice [2] contributed to elevated mRNA abundance of TNF-*α* in the SVF. Alternatively, increased TNF-*α* mRNA abundance may have resulted from increased macrophage infiltration into adipose tissue of ACE2 chimeric mice. This possibility is unlikely since other markers of M1 macrophage polarization, such as CCR2, were not increased in the SVF from ACE2 chimeric mice.

It should be noted that effects of leukocyte ACE2 deficiency to promote F4/80 mRNA abundance in SVF of high fat-fed mice, while significant, were relatively modest (2-fold increase in F4/80 mRNA abundance in ACE2-deficient chimeras). Previous investigators have used bone marrow transplantation with creation of chimeric mice to examine effects of CCR2 deficiency in leukocytes on F4/80 expression in adipose tissue of high-fat-fed mice. Leukocyte deficiency of CCR2, the major receptor for the chemokine MCP-1, resulted in a 50% reduction in F4/80 expression in adipose tissue [29]. Thus, the magnitude of the effect of ACE2 deficiency to promote F4/80 mRNA abundance is consistent with other leukocyte chimeric manipulations.

 To our knowledge, this is the first report that deficiency of leukocyte ACE2 can modulate the development of glucose intolerance in obese mice. Moreover, the magnitude of effect of ACE2 deficiency in bone-marrow-derived cells to promote glucose intolerance in HF mice was most likely under-estimated in the present study due to the pronounced glucose intolerance present in irradiated 4 month HF-fed mice. Indeed, impaired glucose tolerance in chimeric mice lacking ACE2 in leukocytes was supported by increased blood concentrations of glycosylated hemoglobin, a more stable blood predictor of type 2 diabetes [30]. Since plasma insulin concentrations in the present study were not influenced by leukocyte ACE2 deficiency, then these results suggest that the degree of glucose intolerance in chimeric ACE2-deficient mice was not sufficient to further augment hyperinsulinemia in chronic 4 month HF-fed mice. Indeed, plasma insulin concentrations in the present study (>8 ng/mL) in 4 month HF-fed mice were greater than those observed in 2 month HF-fed mice (2 months, 5 ng/mL [31]). Importantly, glucose intolerance was slightly augmented in chimeric mice lacking ACE2 in leukocytes even though these mice exhibited a similar level of obesity compared to wild type controls. In comparison to other studies examining leukocyte deficiency of proteins known to regulate glucose homeostasis, previous studies demonstrated that deficiency of IL-10 [32], leptin [33], toll 4 receptors [34], or PAI-1 [35] had no effect on glucose tolerance in HF-fed mice. Thus, given negative results from leukocyte deficiency of these well-known regulators of glucose homeostasis, it is interesting that deficiency of ACE2 in leukocytes modestly impaired glucose homeostasis in obese mice.

A limitation of this study is that the relative role of AngII versus Ang-(1–7) in the effects of bone marrow ACE2 deficiency on adipose tissue inflammation and glucose homeostasis was not defined. Previous studies demonstrated that mas receptor deficiency in FVB/N mice resulted in marked changes in lipid profiles and glucose homeostasis [36]. In addition, chronic infusion of Ang-(1–7) to fructose-fed rats reduced fasting insulin levels and enhanced insulin signaling pathways (IRS-1/PI3K/Akt) in liver, skeletal muscle, and adipose tissue [37]. An elegant study by Santos et al. utilized a transgenic Sprague-Dawley rat to overexpress Ang-(1–7) systemically and found that plasma triglyceride and cholesterol levels were decreased and whole body insulin sensitivity was enhanced [38]. This group also noted that Ang-(1–7) significantly increased adiponectin levels in rat adipocytes [38]. These results suggest that reductions in Ang-(1–7) may have contributed to the observed effects of leukocyte ACE2 deficiency. Alternatively, since AngII, but not Ang-(1–7), increased adhesion of monocytes to endothelial cells, [2] then AngII may have mediated effects of chimeric ACE2 deficiency. Further studies are needed to clarify the role of the AngII/Ang-(1–7) balance in effects of ACE2 deficiency on adipose inflammation and glucose homeostasis in obese mice. 

## 5. Conclusions

In conclusion, deficiency of ACE2 in leukocytes modestly promoted inflammation in the stromal vascular fraction from visceral adipose tissue and augmented glucose intolerance in mice with diet-induced obesity. Future studies should address the role of ACE2 in inflammation of human adipose tissue and type 2 diabetes.

## Figures and Tables

**Table 1:** Characteristics of chimeric HF-fed mice transplanted with *Ace2^+/y^* or *^−/y^* bone marrow.

	*Ac* *e*2^+/*y*^	*Ac* *e*2^−/*y*^
Body weight (g)	45 ± 1	46 ± 1
Body fat by DEXA (%)	40 ± 1	39 ± 1
Serum cholesterol (mg/dL)	227 ± 9	240 ± 12
Serum triglycerides (mg/dL)	91 ± 9	77 ± 8
Serum NEFAs (mEq/L)	4.3 ± 0.3	4.2 ± 0.4
Plasma renin (ng/mL)	8 ± 1	5 ± 1
Plasma AngII (pg/mL)	243 ± 50	154 ± 35
Serum ACE activity (nmol/mL/min)	742 ± 77	686 ± 96

Data are mean ± SEM from *N* = 3–11 mice/donor genotype.

**Table 2:** Primers used in this study.

Gene	Primers
F4/80	Forward 5′-CTTTGGCTATGGGCTTCCAGTC-3′ Reverse 5′-GCAAGGAGGACAGAGTTTATCGTG-3′
TNF-*α*	Forward 5′-CCCACTCTGACCCCTTTACTC-3′ Reverse 5′-TCACTGTCCCAGCATCTTGT-3′
TNF-*α* receptor	Forward 5′-CAGTCTGCAGGGAGTGTGAA-3′ Reverse 5′-CACGCACTGGAAGTGTGTCT-3′
CCR2	Forward 5′-AGAGAGCTGCAGCAAAAAGG-3′ Reverse 5′-GGAAAGAGGCAGTTGCAAAG-3′
CCL2	Forward 5′-CCTGCTGCTACTCATTCACC-3′ Reverse 5′-TGTCTGGACCCATTCCTTCT-3′
IL-1beta	Forward 5′-ATCTGGGATCCTCTCCAGCCAAGC-3′ Reverse 5′-AAAGGTTTGGAAGCAGCCCTTCAT-3′
PAI-1	Forward 5′-ACTGCAAAAGGTCAGGATCG-3′ Reverse 5′-ACAAAGGCTGTGGAGGAAGA-3′
Mgl-1	Forward 5′-ATGATGTCTGCCAGAGAACC-3′ Reverse 5′-ATCACAGATTTCAGCAACCTTA-3′
IL-4	Forward 5′-AGAAGGGACGCCATGCACGG-3′ Reverse 5′-ATGCGAAGCACCTTGGAAGCCC-3′
YM-1	Forward 5′-GACCTGCCCCGTTCAGTGCC-3′ Reverse 5′-TGCCAGTCCAGGTTGAGGCCA-3′
Mrc-1	Forward 5′-AGGGCAAGCTGCAAGCAGCA-3′ Reverse 5′-CCCACTGCCAACCACTGCGT-3′
